# A retrospective analysis of the utility of endocervical curettage in screening population

**DOI:** 10.18632/oncotarget.15658

**Published:** 2017-02-23

**Authors:** Yan Song, Yu-Qian Zhao, Ling Li, Qin-Jin Pan, Nan Li, Fang-Hui Zhao, Wen Chen, Xun Zhang, You-Lin Qiao

**Affiliations:** ^1^ Department of Pathology, National Cancer Center, Cancer Hospital, Chinese Academy of Medical Sciences & Peking Union Medical College, Beijing, China; ^2^ Department of Cancer Prevention and Treatment, Sichuan Cancer Hospital & Institute, Sichuan Cancer Center, School of Medicine, University of Electronic Science and Technology of China, Chengdu, China; ^3^ Department of Epidemiology, National Cancer Center, Cancer Hospital, Chinese Academy of Medical Sciences & Peking Union Medical College, Beijing, China; ^4^ Department of Gynaecology, National Cancer Center, Cancer Hospital, Chinese Academy of Medical Sciences & Peking Union Medical College, Beijing, China

**Keywords:** cervical intraepithelial neoplasia and cancer, screening, endocervical curettage, cytology, histopathology

## Abstract

The performance of endocervical curettage (ECC) is a dispute in population screening programs. Data of 3,460 women referred to colposcopy examination and had completed pathological results in the Shanxi Province Cervical Cancer Screening Study I and II were reviewed. Among them, 0.6% and 2.7% women were identified as the histopathological confirmed high-grade squamous intraepithelial lesion or worse (HSIL+) by ECC alone or both ECC and quadrants biopsy respectively. Age, cytology, and colposcopy impression are the impact factors for the HSIL+ yield of ECC (*P*<0.05). The age-adjusted odds ratio for cytology and colposcopic impression were 5.283 (95%CI: 3.989-6.997) and 3.609 (95%CI: 2.910-4.476) respectively. In low-grade squamous intraepithelial lesion cytology and abnormal colposcopy, no additional HSIL+ was found by ECC. In low-grade squamous intraepithelial lesion cytology but normal colposcopy, the additional yield was 0.6%, 0.8% and 1.1% for the three age groups respectively. In high-grade squamous intraepithelial lesion or worse cytology, the additional HSIL+ yield by ECC ranged between 1.4% and 6.6%. We conclude that the performance of ECC increases with age, the severity of cytology, and colposcopic impression. For women 35 years and older, ECC should be performed if the cytological finding is high-grade or worse in cervical cancer screening program.

## INTRODUCTION

In the recently published cancer statistic in China, the estimated number of new cervical cancer cases among Chinese women is about 8 times of the cases in the U.S [1, 2]. To improve the government implemented cervical cancer screening program, in which 10 million 35-64 years-old women living in rural areas would be screened every year by county level hospitals, more efficient screening procedure, more experienced gynecologists and pathologists are in need. In current guideline of cervical cancer screening, women with abnormal results should be referred to colposcopy, such as human papillomavirus (HPV) positive with atypical squamous cells of undetermined significance (ASC-US) cytology, or low-grade squamous intraepithelial lesions or worse (LSIL+) cytology alone. Histopathology specimens including biopsy and endocervical curettage (ECC) might be taken under colposcopy. The histology diagnosis is the gold standard to define high-grade squamous intraepithelial lesions or carcinomas (HSIL+) cases [3]. However, due to the lack of enough experienced clinicians and qualified histopathologists, the sampling and diagnose for biopsy and ECC remain obstacles to improving the quality of the program. It has been reported that random biopsy could increase the HSIL+ yield in women with high-grade cytology [4, 5]. The current guideline [6] prefer endocervical sampling for non-pregnant women with LSIL cytology if the colposcopic examination is inadequate or no lesion is identified; and it is acceptable if the colposcopy is adequate and a lesion is present. To perform ECC or not and the efficacy of ECC remains a question in real world population screening. Firstly, ECC is difficult to perform in patients with a stenotic cervix or in menopausal women. Secondly, the inter-observer agreement in the interpretation of ECC specimens is poor. For specific diagnoses, cases interpreted as normal or high-grade dysplasia demonstrated greater agreement than those interpreted as low-grade dysplasia. Individual pathologists' comparison κ values ranged from 0.31 to 0.80, as well as false-positive and false negative results as high as 30% and 50%, respectively [7]. Additionally, ECC is an uncomfortable procedure and has been rated by patients as a 5.8 on a visual analog scale of 0 to 10 in prior studies [8].

Less debate regarding the performance of ECC in unsatisfactory colposcopy would increase the yield of HSIL+ [7]. However, controversy remains of the usefulness of ECC in evaluating women who have abnormal cervical cytology under satisfactory colposcopy. Data are needed to further define the role of ECC in evaluating women who have cervical disease. In our study, data from the Shanxi Province Cervical Cancer Screening Study (SPOCCS) I and II during June 1999 to July 2002 were reviewed to estimate the utility of ECC.

## MATERIALS AND METHODS

### Subjects

**Figure 1:**
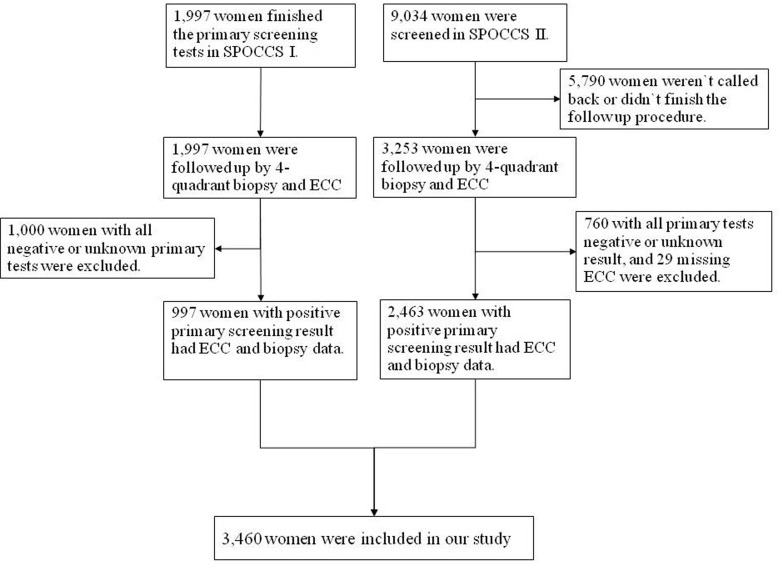
Flowchart of Data Inclusion

11,031 women were screened in the SPOCCS I and II during June 1999 to July 2002. The eligible women were not pregnant currently and had no history of cervical cancer, pelvic radiation, or hysterectomy. For SPOCCS I and II, all women underwent physician-sampling HPV DNA test (Hybrid Capture 2, Digene, Corp), liquid-based cytology (LBC) and visual inspection with 5% acetic acid staining (VIA). For SPOCCS I, all women have had four-quadrant biopsies and ECC under colposcopy regardless of the primary screening results, while women with positive VIA, high-risk HPV or a positive LBC test (ASC-US or worse) were referred to four-quadrant biopsies and ECC in SPOCCS II. A demographic questionnaire was collected by face-to-face interview before clinic examination at baseline. The Institutional Review Boards for human research subjects of both Cleveland Clinic Foundation and CHCAMS approved the two studies. The inclusion flowchart is shown in Figure 1.

### Liquid-based cytology

Specimens for LBC were obtained with a plastic spatula and an endocervical brush and they were placed in transport medium. LBC was prepared *via* the ThinPrep method (Cytyc Corp., Boxborough, MA) and was interpreted using the Bethesda classification system: negative for intraepithelial lesion or malignancy (NILM), ASC-US, atypical glandular cells (AGC), LSIL,, high-grade squamous intraepithelial lesions (HSIL), atypical squamous cells cannot exclude HSIL (ASC-H), and squamous cell carcinoma (SCC). Cytologists from the Cancer Hospital, Chinese Academy of Medical Sciences (CHCAMS) were blinded to other screening tests. Cytologists at the Cleveland Clinic reviewed all abnormal slides and 5% of the normal slides. No significant differences in diagnoses were seen between the Beijing and the Cleveland Clinics.

### Colposcopy and biopsy

Colposcopy was considered satisfactory if the entire squamocolumnar junction could be observed. The cervix was divided visually into four quadrants by lines drawn from 12 to 6 o'clock and from 3 to 9 o'clock positions. Each quadrant of the cervix was graded separately as normal (no visible lesions), low-grade squamous intraepithelial lesion (LSIL) suggestive of HPV or cervical intraepithelial neoplasia 1 (CIN1), high-grade squamous intraepithelial lesion (HSIL) suggestive of intraepithelial neoplasia 2 or 3 (CIN2 or 3), or invasive cervical cancer. All abnormal colposcopic impression by colposcopy was biopsied. If colposcopic examination showed no lesions in any quadrant, a random biopsy was obtained at the squamocolumnar junction in four quadrants at the 2, 4, 8, or 10 o'clock positions, respectively. It was acceptable to take more than one biopsy per quadrant depending on the colposcopic impression. ECC was performed with a Kevorkian curette.

### Pathological diagnosis

The histological diagnosis was based on the consensus diagnosis of 2 pathologists in CHCAMS. In the case of discordant diagnosis, the final diagnosis was based on the assessment of a third pathologist. The final diagnosis was based on the worst biopsy obtained. The biopsies were also sent to the Cleveland Clinic for a similar review. Pathologists were blinded to the results of other tests. No significant differences in diagnosis were found among pathologists from CHCAMS and Cleveland Clinic.

### Data management and statistical analysis

The file copies of all medical records, including sample collection, biological test results, personal questionnaires, were kept in a personal file for every woman. Double entry validation and logical consistency check were performed. SPSS 23.0 were used for data analysis. Mean and the standard deviation was calculated for continuous variables, such as the age at screening, at menarche, and at sex debut. A proportion was calculated for a categorical variable, such as marriage status and tobacco use. Women were stratified by age (< 34, 35-39, 40-44, 45-49, ≥50), cytology (NILM, ASC-US/AGC, LSIL, HSIL/ASC-H/SCC), HPV status (positive, negative), VIA (normal, abnormal, cancer) and colposcopic impression (normal, LSIL, HSIL, cancer). The Cochran-Armitage trend test was used to detect the trend of HSIL+ yield by ECC along with the increasing of age group, severity of cytology, VIA, and colposcopy impression. Binary logistic regression was used to detect the risk factors and the odd ratios (OR) with 95% confidence intervals (95%CI). Age, cytology, HPV status, VIA and colposcopy impression were included as candidate factors. The pathology finding of ECC was the dependent variable. *P* < 0.05 was considered statistically significant.

## RESULTS

### Participant characteristics

As shown in Figure 1, 5,250 of 11,031 women participating in the screening studies were referred to colposcopy and had four-quadrant biopsy and ECC. Among them, 3,460 women who had at least 1 positive primary screening result and completed pathologic results were included in our data analysis. The mean age was 40.6±4.1 years, with a range of 32 to 52. The average age at menarche was 16.2±1.8 years, and the average age for sex debut was 21.1±2.1 years. The vast majority (3,292, 95.1%) of the women were in a marriage or never smoked (3,212, 92.8%), 92.0% (3,183) of the women had contraception measures, and 79.7% (2,758) of them had sterilization.

### Efficacy of biopsy and ECC in detecting HSIL+

Finally, 482 pathologically confirmed HSIL+ cases were diagnosed either by quadrants biopsy and/or ECC, which count 13.7% of the 3,460 women. As shown in Table 1, 361 (74.9%) cases were found by quadrants biopsy alone, 99 (20.5%) cases were diagnosed by both ECC and quadrants biopsy, and 22 (4.6%) cases were found by ECC alone. 121 HSIL+ cases were found by ECC in total, which counts 3.5% (121/3,460) of all the ECC specimens, 0.6% (22/3,460) of all women would be identified as HSIL+ on their ECC alone and 136 or 137 ECC samples would be needed to obtain one additional HSIL+ case that missed by quadrants biopsy. 151 (4.4%) of all the ECC specimens were inadequate. 10.6% (16/151) of the unsatisfactory ECC specimens were found as HSIL+ by quadrants biopsy. Of the 16 CIN2+ cases but unsatisfactory ECC, 15 were HPV positive, 10 was HSIL+ cytology, 10 were normal colposcopy impression.

**Table 1:** Histopathology Diagnosis by Quadrants Biopsies and ECC

Histopathology diagnosis by ECC	Histopathology diagnosis by Quadrants Biopsy									
	SCC		HSIL		LSIL		Normal		Total	
	*n*	%	*n*	%	*n*	%	*n*	%	*n*	%
SCC	5	16.1	2	0.5	1	0.2	0	0.0	8	0.2
HSIL	12	38.7	80	18.8	8	1.8	13	0.5	113	3.3
LSIL	0	0.0	19	4.5	14	3.2	14	0.5	47	1.4
Normal	13	41.9	313	73.6	391	89.9	2424	94.4	3141	90.8
Unsatisfactory	1	3.2	15	3.5	18	4.1	117	4.6	151	4.4
Total	31	0.9	429	12.4	432	12.5	2568	74.2	3460	100.0%

The histopathology diagnose for quadrants biopsy were based on the most severe diagnosis among the four quadrants. The last column and last row were the constituent ratio of pathology classification in all women by ECC and biopsy respectively.

### Risk factors of ECC in detecting HSIL+

Statistical significant trends were found for the HSIL+ yield of ECC by the increasing age, severity of cytology, VIA, colposcopy impression (*P*trend < 0.001). As presented in Table 2, age, cytology, and colposcopic impression were the risk factors for whether ECC detected HSIL+. The age-adjusted ORs for cytology and colposcopic impression were 5.283 (95% CI: 3.989-6.997, *P* < 0.001) and 3.609 (95% CI: 2.910-4.476, *P* < 0.001), respectively. No HSIL+ case was found by ECC in women younger than 35 years or negative HPV testing or unsatisfactory cytology diagnosis. Of all HSIL+ cases detected by ECC, 71.9% (87/121) were found in women with HSIL+ cytology, the yield was 20.8%. 37.2% (45/121) were found in women with HSIL or SCC colposcopy impression, and the yield 22.1%.

**Table 2:** Histopathology Diagnosis of ECC Stratified by Clinical Indications

Subgroups		Histopathology diagnosis of ECC								OR (95%CI)	*P*
		HSIL+		LSIL or Normal		Unsatisfactory		Total			
		*n*	%	*n*	%	*n*	%	*n*	%		
Age	≤34	0	0.0	45	91.8	4	8.2	49	1.5	1.332 (1.035, 1.714)	0.026
	35-39	35	2.3	1396	92.4	80	5.3	1511	45.1		
	40-44	40	3.5	1077	93.3	37	3.2	1154	34.4		
	45-49	36	5.9	549	89.9	26	4.3	611	18.2		
	≥50	3	10.3	23	79.3	3	10.3	29	0.9		
Cytology	HSIL+	88	20.2	336	77.1	12	2.8	436	12.6	3.380 (2.554, 4.473)	<0.001
	LSIL	15	2.5	570	93.1	27	4.4	612	18.2		
	ASC-US/AGC	5	0.6	780	94.2	43	5.2	828	24.7		
	Normal	6	0.4	1400	95.0	68	4.6	1474	43.9		
Colposcopy	SCC	14	41.2	17	50.0	3	8.8	34	1.0	1.605 (1.280, 2.243)	<0.001
	HSIL	31	18.2	137	80.6	2	1.2	170	4.9		
	LSIL	38	4.3	792	90.4	46	5.3	876	25.4		
	Normal	37	1.6	2232	94.3	99	4.2	2368	68.7		
VIA	Cancer	7	41.2	10	58.8	0	0.0	17	0.5	1.242 (0.791, 1.951)	0.346
	Abnormal	55	5.4	908	89.7	49	4.8	1012	29.3		
	Normal	59	2.4	2268	93.4	102	4.2	2429	70.2		
HPV	Positive	120	4.9	2221	91.4	88	3.6	2429	70.7	-	0.986
	Negative	0	0.0	944	93.8	62	6.2	1006	29.3		

106 missing data of age and cytology, 16 of them were histopathology confirmed HSIL+ cases. 4 unsatisfactory cytology findings were LSIL/normal histopathology by ECC. 12 unknown/missing colposcopy results, 3 of them were HSIL+. 2 missing VIA, none HSIL+ were found. 25 unknown/missing HPV results, 2 of them were HSIL+ cases.

**Figure 2:**
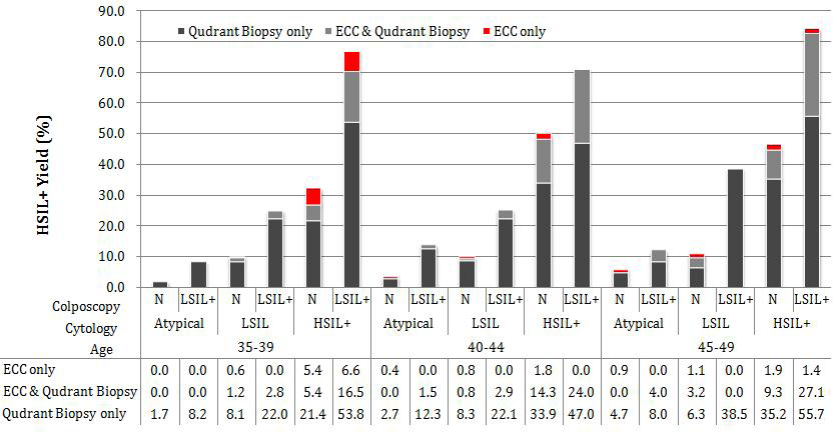
Yield of Histopathology Confirmed HSIL+ cases by ECC and/or Quadrant Biopsy Data for women younger than 35 and older than 50 years were not shown. N: Normal colposcopic impression; cytology Atypical includes ASC-US and AGC; cytology HSIL+ includes ASC-H, HSIL and SCC.

Figure 2 shows detailed HSIL+ yield by ECC and/or quadrants biopsy stratified by age group, cytology, and colposcopy. A limited number of women younger than 35 or older than 50 years (less than 50), and few HSIL+ cases were found by ECC (none for women younger than 35 and 3 for women older than 50), no further discuss those two age groups in Figure 2. In women between 35 and 49 years, if cytology were atypical findings (including ASC-US and AGC), the total yields by ECC for three age groups ranging between 0.0%-4.0%, and the additional HSIL+ yields was low, ranged between 0.0% and 0.9% stratified by normal or abnormal colposcopic impression. If LSIL cytology, the stratified yields of ECC by age and colposcopic findings were higher than atypical cytology, which ranging between 1.8% and 4.3%, except for 45-49 years-old women with LSIL+ colposcopic impression. If colposcopic impression was LSIL+ for LSIL cytology, no additional HSIL+ case was found by ECC. However, if LSIL cytology but normal colposcopy finding, the additional HSIL+ yield was 0.6%, 0.8%, 1.1%, and the number of ECC needed to detect one additional HSIL+ were 157, 110, 86 for women 35-39, 40-44 and 45-49 respectively. In women with HSIL+ cytology, the yield was much higher, ranging between 10.8% and 28.5%, and the additional HSIL+ yield ranged between 1.4% and 6.6%, except for 40-44 years-old women with LSIL+ colposcopic impression that no additional HSIL+ case was found.

## DISCUSSION

The appropriate clinical setting in which ECC should be used is a subject of debate. Some advocate that ECC is unnecessary, due to its poor performance compared with concurrent cervical biopsy specimens. Others use ECC in selected patient populations as an adjunct procedure [7, 8]. In our institution, it has been a routine procedure to perform ECC in all non-pregnant women with low-grade cytological abnormalities, regardless of the adequacy of the colposcopy or the presence of an ectocervical lesion. According to the presented study, the performance of ECC to find HSIL+ cases under some conditions is not satisfactory. Of all the women who have had ECC, only 0.60% of them are benefited from it directly, 136 or 137 ECCs needed to be performed to identify one additional HSIL+ case that may be missed by quadrants biopsy, implying not all women equally benefited and most of them suffered the pain unnecessarily. In our data, it is less necessary to perform ECC among women with atypical or LSIL cytology and positive colposcopy findings.

Age, cytology and colposcopic impression were the association factors to whether ECC could found HSIL+ or not, according to the logistic regression analysis. The HSIL+ yields of ECC increase with age, the severity of cytological abnormality, and the colposcopy findings. Age is a reasonable indication for ECC decision due to the physiological changes of the cervix along with the aging process. In the presented data, no HSIL+ case was found by ECC in women younger than 35 years, which should be attributed to the limited number of younger women included in our study and the lower HISL+ prevalence in this population [9]. The data shows that HSIL+ yield of ECC in ASC-US or AGC or LSIL cytology is low, irrespective of the age group, which is similar to others studies [10–15]. Particularly under the condition that the colposcopic findings are positive, no additional HSIL+ cases were found by ECC alone in our dataset. It implied that in future large population screening programs, women with ASC-US or AGC or LSIL cytology but positive colposcopic impression might be avoided from the unnecessary pain from ECC. However, if the colposcopic impression is negative among women with LSIL cytology, the additional yield is 0.6%, 0.8% and 1.1% for the three age groups. The additional yield may not be satisfactory, but it may be acceptable in older women who had LSIL cytology but negative colposcopic finding. In women older than 35 years with cytological HSIL+, the additional HSIL+ yield by ECC alone should not be ignored, which ranged from 1.4%-6.6% in most cases. This finding suggested that among women with HSIL cytology, ECC is recommended irrespective of the age and colposcopic impressions. A higher detection rate of HSIL+ cases by ECC (4.5%) and similar detection rate among HSIL+ cytology (29.9%) and HSIL+ colposcopy impression (21.2%) women were reported by another retrospective study conducted by Pretorius RG et al [16]. Among the women had ECC, 1.5% (274/18,537) were found HSIL+ lesions by ECC alone. If limiting the number to women older than 35 years only, the yield of ECC alone is 2.4% (189/7,959), which are much higher than that of our data. It could be explained by the different population and referral criterion. Women included in their study were younger with a median age of 32 years old and were patients from clinics. The women referred for colposcopy were ASC-US with positive hrHPV tests or for 2 cytology of ASC-US with negative hrHPV tests 1 year apart, LSIL cervical cytology or 2 negative cytology results with positive hrHPV 1 year apart. While our analysis was based on a referral population from cervical cancer and precancerous screening projects, and any primary result positive women was called back for colposcopy, including abnormal VIA, hr-HPV test positive or LSIL cytology alone, which implied a lower risk of high-grade cervical lesion and cancer in our population.

Strengths of this study are the large sample size, rigorous methodology, and consistency of the biopsy criterion. The worst histopathology diagnosis of cervical four-quadrant biopsy and ECC was taken as final diagnosis and as the gold standard for our data analysis, which could greatly minimize the verification bias in disease detection. In addition to visible lesion-targeted biopsy, normal-appearing quadrant biopsy was performed and under the negative colposcopic impression, the random biopsy was also applied, which decrease misclassification. High level of diagnostic quality control based on three separate pathologist readings also efficiently avoided misclassification. One limitation of our study is the older population, limiting the generalizability to younger populations. Another weakness is the lack of unsatisfactory colposcopy impression data. Many studies suggested that the CIN2+ prevalence in patients with unsatisfactory colposcopy various from 8% to 27% while in satisfactory colposcopy is 1.3% to 12%, much lower than unsatisfactory colposcopy [7, 12, 16, 17]. It suggested that ECC should be performed at unsatisfactory colposcopy.

We concluded that the performance of ECC increases with age, the severity of cytology, and colposcopic impression. For women 35 years and older, ECC should be performed if the cytological finding is HSIL+, especially under normal colposcopy impression in cervical cancer screening program.

## Abbreviations

ASC-US: Atypical Squamous Cells of Undetermined Signification
AGC: Atypical Glandular Cells
ASC-H: Atypical Squamous Cells cannot exclude High-grade
CIN: Cervical Intraepithelial Neoplasia
CHCAMS: Cancer Hospital, Chinese Academy of Medical Sciences
CI: Confidence Interval
ECC: Endocervical curettage
HC2: Hybrid Capture 2
hr-HPV: High risk human papillomavirus
HSIL: High-grade Squamous Intraepithelial Lesions
IARC: International Agency for Research on Cancer
LBC: Liquid Based Cytology
LSIL: Low-grade Squamous Intraepithelial Lesions
NILM: Negative for Intraepithelial Lesion or Malignancy
OR: Odds Ratio
SCC: Squamous Cell Carcinoma
SPOCCS: Shanxi Province Cervical Cancer Screening Study

## References

[1] Chen W, Zheng R, Baade PD, Zhang S, Zeng H, Bray F, Jemal A, Yu XQ, He J (2016). Cancer statistics in China, 2015. CA Cancer J Clin.

[2] Siegel RL, Miller KD, Jemal A (2016). Cancer statistics, 2016. CA Cancer J Clin.

[3] Davey DD, Austin RM, Birdsong G, Buck HW, Cox JT, Darragh TM, Elgert PA, Hanson V, Henry MR, Waldman J (2002). ASCCP patient management guidelines: Pap test specimen adequacy and quality indicators. American journal of clinical pathology.

[4] Pretorius RG, Zhang WH, Belinson JL, Huang MN, Wu LY, Zhang X, Qiao YL (2004). Colposcopically directed biopsy, random cervical biopsy, and endocervical curettage in the diagnosis of cervical intraepithelial neoplasia II or worse. American journal of obstetrics and gynecology.

[5] Song Y, Zhao YQ, Zhang X, Liu XY, Li L, Pan QJ, Shen GH, Zhao FH, Chen F, Chen W, Qiao YL (2015). Random biopsy in colposcopy-negative quadrant is not effective in women with positive colposcopy in practice. Cancer epidemiology.

[6] Wright TC, Massad LS, Dunton CJ, Spitzer M, Wilkinson EJ, Solomon D (2006). consensus guidelines for the management of women with abnormal cervical cancer screening tests. American journal of obstetrics and gynecology.

[7] Zahn CM, Rao LK, Olsen C, Whitworth SA, Washington A, Crothers BA (2011). Reproducibility of endocervical curettage diagnoses. Obstetrics and gynecology.

[8] Driggers RW, Zahn CM (2008). To ECC or not to ECC: the question remains. Obstet Gynecol Clin North Am.

[9] Zhao FH, Lewkowitz AK, Hu SY, Chen F, Li LY, Zhang QM, Wu RF, Li CQ, Wei LH, Xu AD (2012). Prevalence of human papillomavirus and cervical intraepithelial neoplasia in China: A pooled analysis of 17 population-based studies. International journal of cancer.

[10] Miranda AD, Rodriguez R, Novoa DM, Rojas A, Pachon A, DiazGranados CA (2006). The use of endocervical curettage in women with low-grade squamous intraepithelial lesions or atypical squamous cells of unknown significance on Pap smear. Journal of lower genital tract disease.

[11] Williams DL, Dietrich C, McBroom J (2000). Endocervical curettage when colposcopic examination is satisfactory and normal. Obstetrics and gynecology.

[12] Rose JD, Byun SY, Sims SM, Davis JD (2012). The utility of endocervical curettage: does routine ECC at the time of colposcopy for low-grade cytologic abnormalities improve diagnosis of high-grade disease?. American journal of obstetrics and gynecology.

[13] Gage JC, Duggan MA, Nation JG, Gao S, Castle PE (2010). Detection of cervical cancer and its precursors by endocervical curettage in 13,115 colposcopically guided biopsy examinations. American journal of obstetrics and gynecology.

[14] van der Marel J, Rodriguez A, Del Pino M, van Baars R, Jenkins D, van de Sandt MM, Torne A, Ordi J, ter Harmsel B, Verheijen RH, Schiffman M, Gage JC, Quint WG (2015). The Value of Endocervical Curettage in Addition to Biopsies in Women Referred to Colposcopy. Journal of lower genital tract disease.

[15] Poomtavorn Y, Suwannarurk K, Thaweekul Y, Maireang K (2014). Diagnostic value of endocervical curettage for detecting dysplastic lesions in women with atypical squamous cells of undetermined significance (ASC-US) and low grade squamous intraepithelial lesion (LSIL) Papanicolaou smears. Asian Pacific journal of cancer prevention.

[16] Pretorius RG, Belinson JL, Peterson P, Burchette RJ (2015). Which Colposcopies Should Include Endocervical Curettage?. Journal of lower genital tract disease.

[17] Hatch KD, Shingleton HM, Orr JW, Gore H, Soong SJ (1985). Role of endocervical curettage in colposcopy. Obstetrics and gynecology.

